# Arsenite malignantly transforms human prostate epithelial cells *in vitro* by gene amplification of mutated KRAS

**DOI:** 10.1371/journal.pone.0215504

**Published:** 2019-04-22

**Authors:** B. Alex Merrick, Dhiral P. Phadke, Meredith A. Bostrom, Ruchir R. Shah, Garron M. Wright, Xinguo Wang, Oksana Gordon, Katherine E. Pelch, Scott S. Auerbach, Richard S. Paules, Michael J. DeVito, Michael P. Waalkes, Erik J. Tokar

**Affiliations:** 1 Division of the National Toxicology Program, National Institute of Environmental Health Sciences, Research Triangle Park, North Carolina, United States of America; 2 Sciome, LLC, Research Triangle Park, North Carolina, United States of America; 3 David H. Murdock Research Institute, Kannapolis, North Carolina, United States of America; Texas Tech University, UNITED STATES

## Abstract

Inorganic arsenic is an environmental human carcinogen of several organs including the urinary tract. RWPE-1 cells are immortalized, non-tumorigenic, human prostate epithelia that become malignantly transformed into the CAsE-PE line after continuous *in vitro* exposure to 5μM arsenite over a period of months. For insight into *in vitro* arsenite transformation, we performed RNA-seq for differential gene expression and targeted sequencing of KRAS. We report >7,000 differentially expressed transcripts in CAsE-PE cells compared to RWPE-1 cells at >2-fold change, q<0.05 by RNA-seq. Notably, KRAS expression was highly elevated in CAsE-PE cells, with pathway analysis supporting increased cell proliferation, cell motility, survival and cancer pathways. Targeted DNA sequencing of KRAS revealed a mutant specific allelic imbalance, ‘MASI’, frequently found in primary clinical tumors. We found high expression of a mutated KRAS transcript carrying oncogenic mutations at codons 12 and 59 and many silent mutations, accompanied by lower expression of a wild-type allele. Parallel cultures of RWPE-1 cells retained a wild-type KRAS genotype. Copy number analysis and sequencing showed amplification of the mutant KRAS allele. KRAS is expressed as two splice variants, KRAS4a and KRAS4b, where variant 4b is more prevalent in normal cells compared to greater levels of variant 4a seen in tumor cells. 454 Roche sequencing measured KRAS variants in each cell type. We found KRAS4a as the predominant transcript variant in CAsE-PE cells compared to KRAS4b, the variant expressed primarily in RWPE-1 cells and in normal prostate, early passage, primary epithelial cells. Overall, gene expression data were consistent with KRAS-driven proliferation pathways found in spontaneous tumors and malignantly transformed cell lines. Arsenite is recognized as an important environmental carcinogen, but it is not a direct mutagen. Further investigations into this *in vitro* transformation model will focus on genomic events that cause arsenite-mediated mutation and overexpression of KRAS in CAsE-PE cells.

## Introduction

Environmental exposure to arsenic increases the risks of skin, lung, kidney, liver and urinary-bladder cancers [1, 2]. Although the mode of action for arsenic-induced tumors is unclear, many animal and human studies suggest arsenic can act as a carcinogen [3, 4], co-carcinogen [5, 6], or transplacental carcinogen [7]. Arsenite and arsenate, the inorganic tri- and pentavalent forms of arsenic, are considered non-mutagenic in bacterial and human cells [8, 9]. However, arsenic may indirectly cause DNA damage, chromosomal abnormalities, and generation of reactive oxygen species (ROS) like superoxide or hydrogen peroxide [10, 11]. Other transformational effects of arsenic may involve disruption of signaling pathways, miRNA dysregulation, inhibition of DNA repair, or formation of cancer stem cells or polycomb proteins [12–19]. Arsenite and other trivalent species can be acutely cytotoxic by readily binding to intracellular thiols (e.g. GSH) and sulfhydryl sites on macromolecules to inhibit critical biochemical processes [17]. Persistent cytotoxicity from prolonged arsenic exposure and subsequent regenerative proliferation may contribute to carcinogenesis as well [3]. Biotransformation of arsenic involves S-adenosylmethione (SAM), methyltransferases and sulfur redox metabolism so that arsenic-induced interference of methyl-donor pathways could lead to abnormal DNA methylation and histone modification patterns and epigenetic transformation [14, 15, 17, 20–24].

The prostate gland, as part of the urogenital system, is among the many target organs in arsenic carcinogenesis [25–27]. Epidemiologic studies have shown an association of environmental inorganic arsenic exposure with prostate cancer incidence and mortality in the U.S. and abroad [28–30]. Development of immortalized human prostate epithelial cells have greatly advanced prostate cancer research [31, 32]. *In vitro* transformation assays induced by various metals, including arsenic, have provided an invaluable model for examining the multistep events underlying tumor formation (see reviews [19, 33]). RWPE-1 cells [34] were developed as non-tumorigenic, human prostate epithelia for research. Subsequently, our group was able to demonstrate that RWPE-1 cells can be malignantly transformed into the CAsE-PE cell line by continuous exposure after 30 weeks of non-cytotoxic levels of sodium arsenite at 5μM in culture [35]. CAsE-PE cells create tumors when injected into nude mice and demonstrate many *in vitro* characteristics of malignant transformation, including loss of contact inhibition, anchorage-independent growth, resistance to apoptosis, and increased activity of secreted MMP-9 [35, 36].

Early work with CAsE-PE cells reported an increased KRAS expression by northern and western blot with absence of mutations by PCR-RFLP, along with MAP kinase and ErbB-2 activation and androgen independent growth [25, 37]. KRAS mRNA and protein were highly overexpressed in CAsE-PE cells beginning at 12 weeks and increasing over a 30 week period [25]. Initial evaluation showed KRAS overexpression in absence of mutations or proximal DNA methylation changes to the gene body [25]. KRAS knockdown in shRNA experiments can reverse many CAsE-PE malignant properties that include a reduced metalloprotease activity, limited anchorage independent growth, reduced cell proliferation, less invasiveness, and decreased MAPK signaling activities [38]. Other studies using these two cell lines reported epigenetic changes such as transcriptional silencing of ZNF (zinc finger) transcriptional repressors, developmental HOX and protocadherin gene families [39, 40] by focal hypermethylation. Carcinogenesis-related genes such as S100P, HYAL1, NES, and NTM have been similarly shown to have an inverse relationship of DNA methylation to gene expression [41]. Further, KRAS activation in arsenic transformed cells has been associated with dysregulated miRNA expression linked to transcriptional regulation of many downstream processes supporting malignancy [42]. Thus, accumulated evidence suggests increased KRAS expression plays a key role in arsenic-induced malignancy in the CAsE-PE cell model.

KRAS is well-recognized as a transformative factor in prostate cancer [43–47] and gene amplification at KRAS loci (e.g. increased copy number) has also been detected in many tumors including prostate [47–49]. In this study, we hypothesized genomic and transcriptomic analyses and targeted KRAS sequencing could reveal insights into arsenite transformation of RWPE-1 into CAsE-PE cells by looking for sequence variations in KRAS, alterations in gene and pathway expression, and genomic alterations contributing to development of malignancy.

## Results

### Alignment of reads, transcript assembly and differential expression

RNA-seq analysis was performed after polyA selection from three independently grown RWPE-1 and CAsE-PE cultures. Low quality bases at the ends of reads were removed and trimmed according to quality metrics (Phred Scores) and equivalent nucleotide distributions (S1 Fig) as previously described [50]. The total number of sequenced reads after quality-based trimming (S1 Table) ranged from 176–198 million paired end (PE) reads. Reads were aligned to hg19 using TopHat for a >200X total coverage of the transcriptome. The initial CAsE-PE alignments to hg19 were found to average 56.8% and were less than the average 77% of RWPE hg19 alignments. DNA-seq at low level coverage was performed to provide additional data to improve genomic alignment. Nearly 500 million DNA-seq reads were obtained for each cell line that were combined and ultimately assembled into 226,987 contigs, mapped to hg19 and RefSeq, excluding non-repetitive regions. Comparison with hg19 and contigs improved alignments to a mean of 71.6% for CAsE-PE and 82.0% for RWPE cells as shown in S1 Table.

CuffDiff was used to determine differential expression using RefSeq annotation from 46,090 transcripts and isoforms of which there were 7,265 total differences (q-value<0.05; >2-fold). From this total number of differentially expressed genes (DEGs) there were 3,261 up-regulated transcripts and 4,004 transcripts which were downregulated compared to RWPE-1 expression (S2 Table).

### Differential expression and pathway activation by arsenic transformation

**Fig 1:**
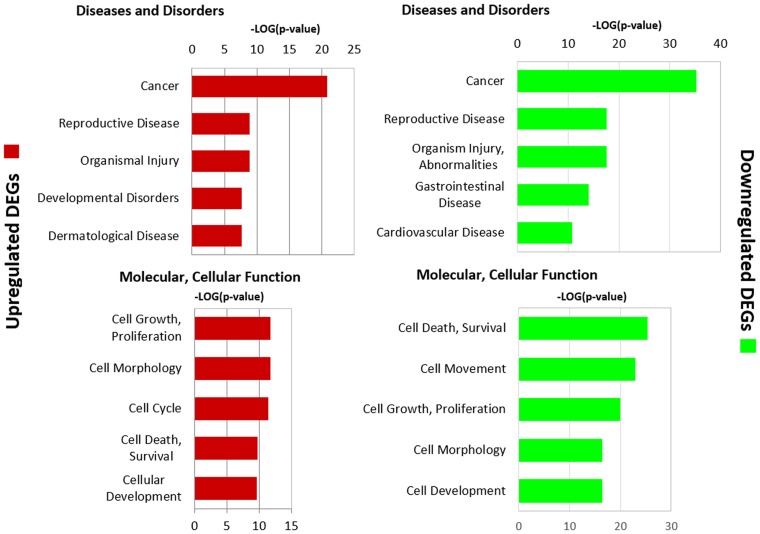
Differentially expressed genes (DEGs) and pathways in arsenite transformed CAsE-PE cells compared to normal RWPE-1 prostate epithelia cells. Expression profiling by RNA-seq showed over 7,000 DEGs at q<0.05 in CAsE-PE cells compared to RWPE-1 cells. DEGs for upregulated genes (red bar graphs) or downregulated genes (green bar graphs) populated pathways that are associated with diseases and health disorders, and changes in molecular and cellular pathways (IPA, Ingenuity Pathway Analysis).

**Fig 2:**
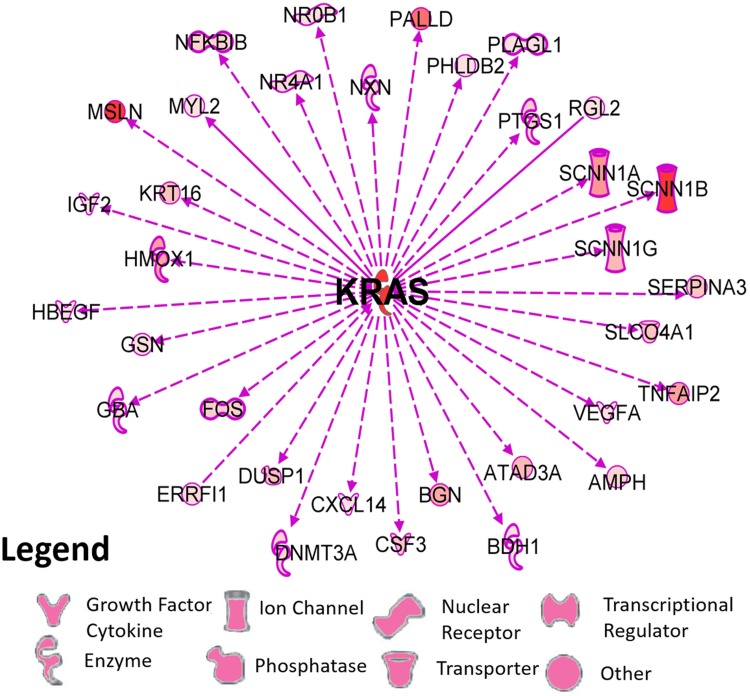
Upregulated genes in CAsE-PE versus RWPE-1 cells that influence KRAS expression. Up-regulated genes were filtered by IPA annotation as either upstream and regulating KRAS (arrow tip towards KRAS) or downstream as KRAS-controlled genes (arrow tip towards gene). Solid lines indicate direct relationship, while dotted lines indicate an indirect relationship. The legend shows symbols representing primary functions of each gene.

The top thirty increased and decreased DEGs are shown in Table 1. Notably, the proliferation gene transcript KRAS at 432-fold increase and the maternally imprinted gene transcript, H19, at 154-fold increase were highly upregulated. PSCA (prostate stem cell antigen), a recognized surface marker of prostate cancer [51], was increased 52-fold (Table 1). Altered molecular functions and disease pathways are displayed in Fig 1. Developmental, morphological and cell growth pathways were altered, as well as changes in reproductive and neoplastic disease pathways that are consistent with prostate malignancy. Fig 2 shows 34 downstream gene-pathway interactions annotated for KRAS (see S3 Table for citations) that involved substantially upregulated transcripts at ≥5-fold. High levels of KRAS were associated with increased expression of several growth factors such as CSF3, IGF2, HBEGF, VEGFA; sodium channel isoforms like SCNN1A, B, and G; transcriptional regulators including FOS, NFKBIB, PLAGL1; and with upregulation of enzymes such as HMOX1, PTGS1 and DNMT3a.

**Table 1:** Top 30 upregulated and downregulated gene fold changes in CAsE-PE versus RWPE-1 cells.

Fold Δ	Transcript ID	Gene Symbol	Entrez Gene Name	Function
959.26	NM_003490	SYN3	synapsin III	synaptic vesicles
432.37	NM_033360	KRAS	KRAS proto-oncogene, GTPase	enzyme
320.77	NM_015568	PPP1R16B	protein phosphatase 1 regulatory subunit 16B	phosphatase
255.16	NM_004121	GGT5_Variant2	gamma-glutamyltransferase 5	enzyme
188.98	NM_022369	STRA6	stimulated by retinoic acid 6	transporter
159.35	NM_033159	HYAL1	hyaluronoglucosaminidase 1	enzyme
154.58	NR_002196	H19	H19, imprinted maternally expressed transcript	imprinted gene, lncRNA
151.47	NM_000507	FBP1	fructose-bisphosphatase 1	phosphatase
119.89	NM_019601	SUSD2	sushi domain containing 2	cytokine receptor
98.69	NM_001190202	CES4A	carboxylesterase 4A	enzyme
86.06	NM_020142	NDUFA4L2	NDUFA4, mitochondrial complex associated like 2	enzyme
85.29	NM_002281	KRT81	keratin 81	structural protein
83.44	NR_052017	MED24	mediator complex subunit 24	transcription regulator
83.01	NM_138567	SYT8	synaptotagmin 8	transporter
78.25	NM_001099781	GGT5_Variant1	gamma-glutamyltransferase 5	enzyme
72.57	NM_001270991	EPGN	epithelial mitogen	growth factor
72.39	NM_130842	PTPRN2	protein tyrosine phosphatase, receptor type N2	phosphatase
67.91	NM_001242767	MTHFD1L	methylenetetrahydrofolate dehydrogenase 1 like	enzyme
67.89	NM_007129	ZIC2	Zic family member 2	transcription regulator
65.52	NM_172084	CAMK2B	calcium/calmodulin dependent protein kinase II beta	kinase
61.26	NM_153480	IL17RE	interleukin 17 receptor E	cytokine receptor
57.39	NM_001958	EEF1A2	eukaryotic translation elongation factor 1 alpha 2	translation regulator
56.66	NM_138768	MYEOV	myeloma overexpressed	tissue invasion
55.13	NR_047690	HYAL1_lncRNA	hyaluronoglucosaminidase 1	enzyme
52.87	NM_005672	PSCA	prostate stem cell antigen	cell growth regulator
52.30	NM_000336	SCNN1B	sodium channel epithelial 1 beta subunit	ion channel
48.60	NR_033861	LINC00514	long intergenic non-protein coding RNA 514	lncRNA
47.85	NR_046224	LINC00659	long intergenic non-protein coding RNA 659	lncRNA
47.52	NM_001171946	SUN1	Sad1 and UNC84 domain containing 1	nuclear envelope
45.58	NR_105044	LOC102546229	uncharacterized LOC102546229	unknown function
-162.16	NM_001001668	ZNF470	zinc finger protein 470	transcription regulator
-163.01	NM_018650	MARK1	microtubule affinity regulating kinase 1	kinase
-167.92	NM_024501	HOXD1	homeobox D1	transcription regulator
-177.01	NM_005602	CLDN11	claudin 11	tight junction protein
-178.56	NM_033518	SLC38A5	solute carrier family 38 member 5	transporter
-179.77	NM_001129891	FAM196B	family with sequence similarity 196 member B	cell growth regulator
-181.54	NM_032682	FOXP1	forkhead box P1	transcription regulator
-185.37	NM_007361	NID2	nidogen 2	ECM protein
-188.33	NM_001010924	FAM171A1	family with sequence similarity 171 member A1	unknown function
-188.57	NM_005103	FEZ1	fasciculation and elongation protein zeta 1	cell growth regulator
-195.20	NM_199320	JADE1	jade family PHD finger 1	transcription regulator
-196.28	NM_000922	PDE3B	phosphodiesterase 3B	enzyme
-197.65	NM_005708	GPC6	glypican 6	transmembrane receptor
-227.31	NM_018043	ANO1	anoctamin 1	ion channel
-237.88	NM_001289861	PER3	period circadian regulator 3	circadian regulator
-246.68	NM_020814	MARCH4	membrane associated ring-CH-type finger 4	enzyme
-249.17	NM_001197294	DPYSL3	dihydropyrimidinase like 3	enzyme
-252.33	NM_001190972	C8orf88	chromosome 8 open reading frame 88	negative translation
-267.80	NM_002403	MFAP2	microfibril associated protein 2	microfibril
-290.47	NM_002523	NPTX2	neuronal pentraxin 2	synaptic vesicles
-305.87	NM_020828	ZFP28	ZFP28 zinc finger protein	transcription regulator
-316.95	NM_152476	ZNF560	zinc finger protein 560	transcription regulator
-337.73	NM_000885	ITGA4	integrin subunit alpha 4	transmembrane receptor
-357.26	NM_170697	ALDH1A2	aldehyde dehydrogenase 1 family member A2	enzyme
-375.46	NR_030299	mir-573	microRNA 573	microRNA
-391.38	NR_036521	ZNF667-AS1	ZNF667 antisense RNA 1 (head to head)	transcription regulator
-513.45	NM_001007026	ATN1	atrophin 1	transcription regulator
-630.89	NM_002535	OAS2	2'-5'-oligoadenylate synthetase 2	enzyme
-683.93	NM_003070	SMARCA2	SWI/SNF related, a2	transcription regulator
-750.77	NM_016608	ARMCX1	armadillo repeat containing, X-linked 1	protein binding

Differentially expressed genes (DEGs) were determined by RNA-seq analysis of arsenic transformed cells (CAsE-PE) compared to the control non-transformed human prostate epithelial cells, RWPE-1. The top 30 upregulated or downregulated gene transcripts are accompanied by their relative fold change (Fold Δ), RefSeq identity, gene symbol, gene description and cellular function.

**Fig 3:**
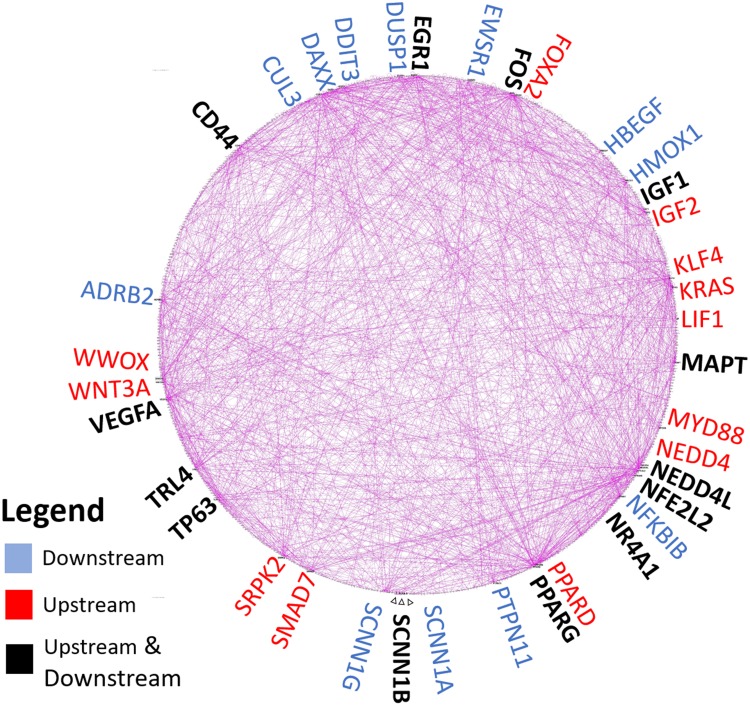
Circular plot of gene connection analysis in CAsE-PE cells. Connections analysis was applied to the top 1000 upregulated DEGs (limit of algorithm) in CAsE-PE vs RWPE-1 cells. All 1000 genes were plotted. IPA (Ingenuity Pathways Analysis) Connections analysis finds annotated relationships among DEGs as participating in regulating (upstream genes) or regulated (downstream genes) relationships. The number of connections were scored for each gene and are summarized in S4 Table. The top 25 upstream and downstream genes are shown in large colored font around the circular plot (downstream genes in blue; upstream genes in red) with bolded (black font) genes having connections for both upstream and downstream relationships. The greater number of connections infers a higher level of regulatory and interactive relationships compared to other genes that are visually indicated by the dark convergence of connections on circle’s rim for specific genes and quantitatively scored by counting the number of upstream and downstream relationships.

Ingenuity (IPA) connectivity analysis was performed for the top 1,000 upregulated DEGs (algorithm limit) to determine the complex regulatory interrelationships of KRAS as displayed in a circular plot (Fig 3). In this analysis, gene-to-gene connections encompass changes in expression, gene and pathway activation, protein-protein interactions, phosphorylation and other interactions (see S4 Table). A greater number of connections for specific genes infers a higher level of biochemical and regulatory importance (darkened convergence of lines on edge of circle). The number of up- or downstream relationships among DEGs were tabulated in S4 Table. Fig 3 shows the top 25 upstream and downstream DEGs in large font at the edges of the circle plot (14 bolded genes in black were common to both lists of up- and downstream relationships). Among these interconnected genes with KRAS were several growth factors, such as IFG1, IGF2, EGR1, VEGFA, HBEGF, transcription factors FOS, FOXA2, TP63, DAXX, DDIT3, and enzymes like NEDD4, NEDD4L, HMOX1, and CUL3, that are consistent with cell proliferation, stress and turnover. DAVID analysis for functionally annotated genes was also performed and showed clustering for EGF-binding domains, cell growth and developmental factors, cellular adhesion factors, Zn finger proteins and cellular membrane changes (S5 Table).

### KRAS mutations and copy number

KRAS is one of the most frequently mutated genes in human cancers. Prior work [25] had used PCR-RFLP to examine codons 12, 13 and 61 for mutations in parent RWPE-1 and arsenite malignantly transformed CAsE-PE cells. These data reported an increase in transcript expression of wild type (wt, as unmutated) KRAS in CAsE-PE cells. Since CAsE-PE cells are malignantly transformed, we decided to re-examine the KRAS genotype in these cells by Sanger sequencing of PCR products from DNA and RNA extracted from each cell type. In normal tissues, KRAS consists of 5 coding exons, exons 1, 2, 3, 4a and 4b (see S2 Fig for structure). A 450bp invariant region includes all exons, except exon 4a and exon 4b. KRAS gene is normally expressed as two splice variants, where the variant 4b is more prevalent in normal cells compared to variant 4a [52]. Mutations in either variant renders them oncogenic, and in human cancers, variant 4a often becomes the more widely expressed KRAS transcript [53].

Initially, we designed primers to examine the genomic sequence of KRAS in both cell types and made several key observations, outlined below. First, exon-specific primers were designed from wt normal sequence of KRAS4a (NM033360) and produced amplicons with sequences aligning to wt KRAS in both cell lines. Similarly, we also designed intronic primers surrounding each exon (exons 1, 2, 3 and 4a) from wt KRAS4a and found Sanger sequences aligned to wt KRAS for RWPE-1, CASE-PE and a normal human prostate DNA sample. Second, we considered the possibility that increased KRAS expression could be related to genomic amplification and designed assays to examine copy number variation (CNV) using wt primers. We found that DNA isolated from RWPE and normal human prostate cells showed two copies of exon 4a and exon 3 as well as two copies of introns 3 and 5 (S3 Fig). These results suggested the presence of normal diploid KRAS alleles in RWPE-1 cells and normal prostate epithelia. However, CAsE-PE cells showed 24 copies of exon 4a, and only one copy of exon 3, intron 3 and intron 5. We interpreted these results, and the wt KRAS exon sequencing data described above, to mean CAsE-PE had one normal KRAS allele (one copy of exon 3, introns 3 and 5) even though qPCR for exon 4a showed 24 copies. We considered that the primer set for exon4a might be detecting two different KRAS alleles, one normal and one aberrant KRAS allele.

Third, the differing copy number of KRAS exons and introns in CAsE-PE cells made us suspect allelic variation of KRAS. To understand the increase in DNA copy number of exon 4a in CAsE-PE cells, experiments were designed for KRAS genomic amplification. To test this hypothesis, we performed PCR on gDNA using various primers sets spanning multiple exons that included exons 1–2, exons 2–3, exons 1-4a, exons 2-4a, and exons 3-4a. Using gDNA, we found amplicons of continuous exons of the expected size for all these regions in CAsE-PE cells only, and not RWPE-1 cells (S4 Fig).

Fourth, Sanger sequencing on the largest KRAS amplicon (exons 1-4a), spanning codons 27–182 in the preceding experiment, showed multiple sequence variants (Table 2) that suggested presence of a mutated KRAS gene allele. Missense mutations were present at codons 59 and 132 as well as twenty synonymous mutations. A redesign of primers was necessary to obtain a complete sequence of exon 1 (S6 Table) and sequence results showed a missense mutation at codon 12 (p.G12S). A table of all KRAS mutations is shown in Table 2. The Catalogue Of Somatic Mutations in Cancer (COSMIC) database was reviewed for annotated mutations. For the three missense mutations, two had COSMIC identifications as ‘oncogenic’ at codons 12 and 59 while the mutation at codon 132 has not yet been annotated. The majority of KRAS mutations were silent coding mutations for which three were identified in the COSMIC database at codons 38, 42 and 73. For mutation types within KRAS, we noted more transition mutations (18) than transversions (5). Comparison of the CAsE-PE mutated allele sequence with the pseudogene, KRASP1, shows KRAS mutations were almost completely unique from the pseudogene (S7 Table) and suggests results were not confounded by KRASP1.

**Table 2:** KRAS mutations in CAsE-PE cells.

Mutation	Mutation	Mutation ID	Mutation Type	FATHMM	Mutation Type	A	G	C	T
(CDS)	(AA)	(COSM No.)	(AA)						
c.34G>A	p.G12S	COSM1152506	Substitution–Missense	Pathogenic (score 0.98)	Transition		X		
c.90C>T	p.D30D		Substitution–coding silent		Transition			X	
c.102A>T	p.P34P		Substitution–coding silent		Transversion	X			
c.105A>G	p.T35T		Substitution–coding silent		Transition	X			
c.114T>C	p.D38D	COSM2202568	Substitution–coding silent	Pathogenic (score 0.75)	Transition				X
c.126G>A	p.K42K	COSM2202566	Substitution–coding silent	N/A	Transition		X		
c.175G>A	p.A59T	COSM1562187	Substitution–Missense	Pathogenic (score 0.98)	Transition		X		
c.219G>A	p.R73R	COSM2202564	Substitution–coding silent	Pathogenic (score 0.82)	Transition		X		
c.309T>A	p.V103V		Substitution–coding silent		Transversion				X
c.327A>G	p.V109V		Substitution–coding silent		Transition	X			
c.345A>G	p.G115G		Substitution–coding silent		Transition	X			
c.351A>G	p.K117K		Substitution–coding silent		Transition	X			
c.359T>C	p.D119D		Substitution–coding silent		Transition				X
c.381A>G	p.T127T		Substitution–coding silent		Transition	X			
c.393G>A	p.Q131Q		Substitution–coding silent		Transition		X		
c.396C>G	p.D132E		Substitution—Missense		Transversion			X	
c.405A>G	p.R135R		Substitution–coding silent		Transition	X			
c.414A>G	p.G138G		Substitution–coding silent		Transition	X			
c.420T>A	p.P140P		Substitution–coding silent		Transversion				X
c.423T>C	p.F141F		Substitution–coding silent		Transition				X
c.429A>G	p.E143E		Substitution–coding silent		Transition	X			
c.432A>C	p.T144T		Substitution–coding silent		Transversion	X			
c.438A>G	p.A146A		Substitution–coding silent		Transition	X			

Mutations in KRAS for CAsE-PE cells were determined by Sanger sequencing and confirmed by Roche 454 sequencing. The specific nucleotide change from wild type sequence is described from the CDS start site (e.g. c.34G>A) accompanied by the amino acid (AA) change (e.g. p.G12S). COSMIC database annotations were noted if present, including the type of mutation (e.g. substitution; transition) and affected nucleotide (marked by X) in far-right columns. FATHMM is the Functional Analysis through Hidden Markov Models assigning relativity pathogenicity scores to point mutations (See, http://fathmm.biocompute.org.uk/). Further details are described in the text.

**Fig 4:**
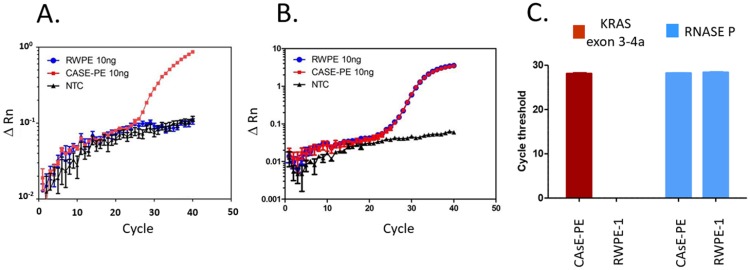
qPCR analysis of RWPE-1 and CASE-PE cells for KRAS exon-3 to exon-4a amplicon. Each point in panel A is the mean ± SEM of 4 replicates per sample at 10ng DNA input. RNAse P in Panel B was multiplexed to show equivalent DNA input per sample. NTC is the non-template control. Panel C shows the mean ± SEM for cycle threshold (Ct) of 4 replicates for CAsE-PE and RWPE-1 cells for KRAS and RNAseP from data in Panels A and B.

Fifth, increased copy number of mutant KRAS allele was confirmed by a qPCR assay using primers crossing over exon3 and exon4A. qPCR included RNAse P as a housekeeping gene to demonstrate equivalent DNA input. Fig 4 shows plots of the ΔRn (normalized reporter value) against the PCR cycle for the KRAS crossover amplicon (Panel A) and RNAse P (Panel B). The Ct (cycle threshold above background) for RWPE-1 and CAsE-PE samples are shown in Panel C. The CAsE-PE samples show clear amplification curves for KRAS exon3-4a amplicon formation while there was no product found in the RWPE-1 samples or the non-template controls. Amplification of RNAse P shows equivalent amount of DNA input into the assay as an internal control.

**Fig 5:**
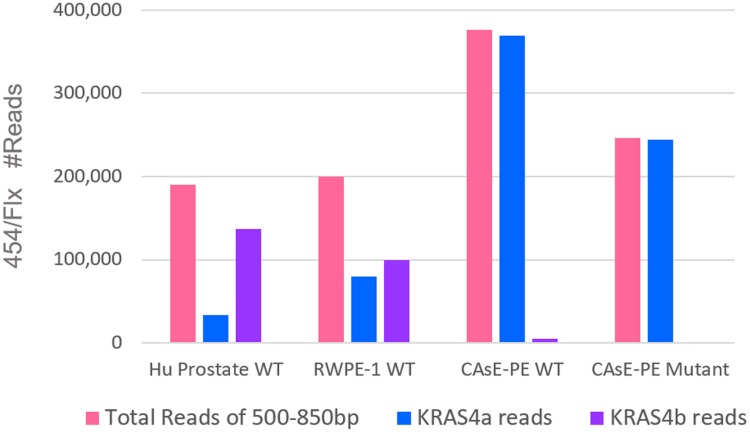
KRAS isoforms, KRAS4a and KRAS4b were measured by 454 sequencing. Sequences were filtered for reads between 500 and 850 bp and then aligned to the two KRAS splice variants. Those reads containing the mutated bases in Table 2 were described as ‘mutant KRAS’ and those reads without those base changes were described as ‘wild type KRAS’. See Methods for further details.

Finally, we wanted to determine the relative proportion of transcripts for KRAS variants 4a and 4b. RNA was isolated from RWPE-1 cells, CAsE-PE cells and normal human prostate cells and cDNA was reverse transcribed with an oligo dT primer. PCR primers were designed from wt KRAS as well as mutant KRAS derived from the mutated allele sequence. Amplicons were produced (S5 Fig) for all three cell types for wt KRAS, but only CAsE-PE produced a mutant amplicon. Indexed libraries were constructed from amplicons from each sample for 454 sequencing. Sequences were filtered for reads between 500 and 850 bp and then aligned to the two splice variants. Those reads containing mutated bases as described in Table 2 were designated as ‘mutant KRAS’ and those reads without those base changes were designated as, ‘wild type KRAS’. Cumulative read counts for 454 sequencing (S8 Table) are shown in Fig 5. The normal human prostate cells had a 4:1 higher proportion of wt KRAS variant 4b compared to variant 4a. Slightly more KRAS4b to 4a reads (55:45) were observed for RWPE cells. Only CAsE-PE produced amplicons from mutant primers that were almost completely mutant variant KRAS4a transcript reads with only a few reads detected for mutant variant KRAS4b transcripts. By comparison, CAsE-PE RNA produced amplicons from wt KRAS primers with a high proportion of mutant KRAS4a variant and lesser amounts of reads aligning to the wt KRAS4b variant. In summary, both RWPE and CAsE-PE expressed wt KRAS4a and KRAS4b but only CAsE-PE cells over-expressed a mutated, oncogenic form of KRAS primarily comprised of variant KRAS4a.

## Discussion

A major finding of this study was the highly mutated nature of a KRAS allele as well as its substantial overexpression. Missense mutations at codon 12 (p.G12S) and codon 59 (p.A59T) are both pathogenic as documented in the COSMIC database of human cancer mutations [54]. Codon 12 and codon 59 KRAS mutations are most commonly found in the large intestine tumors and to a lesser extent in lung, hematopoietic and stomach tumors. The sequence variant at codon p.D132E has not yet been reported for KRAS in human tumors. It is notable the other sequence variants result in silent mutations, most of which have not been annotated. KRAS mutations likely accumulate through clonal selection over months of arsenite exposure. Importantly, the concurrence of wt and amplified mutant KRAS DNA in CAsE-PE cells is consistent with the genomic aberration, ‘mutant allele specific imbalance’, known as MASI, that is frequently observed for oncogenes in many primary tumors [55]. The incidence of KRAS MASI genotypes range from about 12 to 18% of clinical cases that include colorectal [56, 57] and lung [58], and pancreatic [59] cancers involving mutations in codons 12 or 13. One study that examined over 400 human tumors, cell lines and xenografts of various tumor types reported that KRAS MASI was often present in primary tumors and malignantly transformed cell lines and that the combination of oncogene mutation, copy number gains and MASI may have a greater role in development and maintenance of malignancy than any individual alteration alone [47]. A more recent study involving more than 1,110 KRAS mutant tumors found an incidence of allelic imbalance at 55% [60]. Even though malignantly transformed cells may contain multiple gene mutations, sequencing studies suggest that mutations in driver oncogenes are often mutually exclusive, meaning the actions of one oncogene may predominate over others [61]. In recent work, knockdown of KRAS expression in CAsE-PE cells greatly reduced several malignant characteristics including anchorage independent growth and metalloprotease activity [38]. Thus, the high level of KRAS gene amplification, transcript expression and activating mutations suggest it is the lead oncogene driving the malignantly transformed CAsE-PE phenotype.

We also measured relative expression of the two splice variants, KRAS4a and KRAS4b, based on long 454 reads. Read sequences containing all detected mutations were called ‘mutated KRAS’ and if no changes were observed from the KRAS invariant sequence, reads were designated ‘wild type KRAS’. KRAS4b is the major splice variant in normal tissues and organs, while the expression of KRAS4a variant is proportionately less and varies from moderate (e.g. colon, pancreas), to low (e.g. lung, prostate) to undetectable (e.g. brain, heart) amounts [62]. Here, normal human prostate early passage epithelia expressed KRAS transcripts (Caucasian donor) had a higher proportion of wt KRAS variants 4b over 4a at a 4:1 ratio. By comparison, RWPE-1 cells had a slightly greater amount of 4b over 4a variants which were both wild type. However, reads for mutant KRAS4a transcripts in CAsE-PE cells were at a much higher ratio at >100:1 ratio compared to either wt KRAS4b reads or the few detectable mutant KRAS4b reads. This is consistent with others, reporting that KRAS4a is more highly expressed in many cancers [53, 63, 64] compared to KRAS4b. The relative variant expression may be important since different biological activities of variants 4a or 4b may uniquely contribute to the malignant transformation process and maintenance of a malignant phenotype. Given the growth-inhibitory actions of wt KRAS in tumors [65, 66], CAsE-PE cells fit an established pattern of increased copy number gains of mutated KRAS resulting in allelic imbalance (MASI) that counteracts the presence of any wt KRAS transcripts. Whether continued exposure of CAsE-PE cells to arsenite might eventually lead to complete loss of wt KRAS has yet to be determined.

How mutations arise in the parent RWPE-1 cells during arsenite exposure and malignant transformation is not clear. Arsenite is recognized as an indirect carcinogen, acting by a combination of redox reactions, DNA damage by oxygen and nitrogen radicals, interference of DNA replication and repair, and epigenetic changes [67–70]. Specifically, arsenite may produce multiple reactive oxygen and nitrogen species that result in DNA damage and mutations [71, 72]. Among various DNA lesions formed by oxidative stress, 8-oxo-guaninine is one of the most common and well-studied oxidative species which frequently results in G→T transversions [73, 74]. Here, we primarily observed purine transitional mutations in KRAS and 4 transversions which were generally silent. The p.G12S substitution at codon 12 was a G>A transition mutation as was the p.A59T substitution at codon 59. These data suggest the mechanism for arsenite-induced mutations is likely more complicated than oxidative DNA damage alone in the CAsE-PE malignant transformation model.

**Fig 6:**
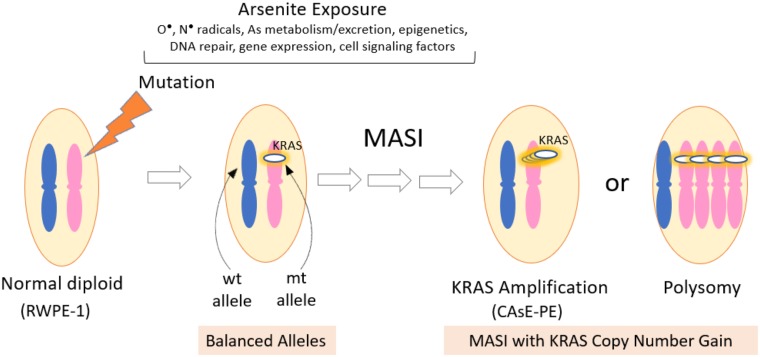
Overview of arsenic-induced malignant transformation in CAsE-PE cells. Prolonged, continuous exposure of RWPE-1 prostate epithelial cells to 5 μM sodium arsenite results in the malignantly transformed CAsE-PE cell line [35]. Our observations suggest that arsenite-induced changes in various cellular factors produce the CAsE-PE cell line with a mutant (mt) KRAS allele and a wild type (wt) allele, in a process termed, mutant allele specific imbalance (MASI). The mt KRAS allele becomes amplified in what is likely a multi-step process (multiple arrows) for a copy number gain that does not involve polysomy. RNA-seq showed a substantial gene expression differences between RWPE-1 and CAsE-PE cells. Figure adapted from Yu et al [55]. Please see text for further explanation.

The molecular processes for malignant transformation of RWPE-1 cells into CAsE-PE cells after prolonged 5 μM arsenite exposure are not completely clear [35]. A 5 μM concentration of arsenite was selected because of minimal cytotoxicity [36] and absence of oxidative DNA damage [75] in RWPE-1 cells. In other studies, when RWPE-1 cells were exposed to low concentrations of arsenite at 100 pg/ml for six months, changes in expression of epigenetic regulatory genes were observed along with global changes in DNA methylation and histone modifications, suggesting epigenetic alterations may also contribute to malignant arsenite transformation [76]. What we do report in this study is formation of an arsenite-induced, oncogenic mutated KRAS allele that becomes amplified in the CAsE-PE line. Increased copy number or gene amplification is a common observation in different types of human tumors. For example, high copy numbers of ERBB2, TOP2A, CCND1, EGFR and MYC are observed in many colorectal cancers [77]. Wide regions of DNA (kilobases to megabases) containing these oncogenes can be organized as extrachromosomal copies (double minutes), as tandem repeats within a chromosome or may be distributed at various sites within the genome [78]. KRAS amplifications has been reported in various malignancies, primarily in pancreatic [59, 79], colorectal [80, 81] and lung [58, 82] tumors, but also have been noted in gastric [83], ovarian [84] and endometrial [85] malignancies, but less frequently in prostate cancers [46]. In our study, the data did not suggest either amplification of large chromosomal portions of DNA encapsulating the KRAS gene, or chromosome duplication (e.g. polysomy); rather the data indicate an insertion of a fully processed mutated KRAS transcript into the genome resulting in increased copy number within CAsE-PE cells. The mechanism of how this type of amplification might occur is currently under investigation. Fig 6 provides a summary of arsenite-induced malignant transformation in RWPE-1 cells beginning with DNA damage and mutation resulting in mutant KRAS allele imbalance and then KRAS gene amplification in CAsE-PE cells.

Expression analysis by RNA-seq shows As transformation activates pathways and cellular processes supporting cell proliferation for tumor development in CAsE-PE cells [29]. Prominent upregulation of KRAS transcripts stands out as a leading driver of malignant transformation in CAsE-PE cells because of its known role in GTPase-mediated cell signaling and proliferation [86]. RAS proteins are master regulators of multiple downstream signaling cascades [87]. Some of the most proximal pathways for KRAS activation involve Raf-MEK-ERK (MAPK) and PI3K/Akt signaling [88]. In our study, we filtered elevated CAsE-PE transcripts and found 34 genes that had annotated interactions with KRAS (S3 Table), including growth factor genes like CSF3, HBEGF, IGF2 and VEGFA and genes for transcriptionally active proteins such as FOS, NR0B1, NR4A1 and PLAGL1. Establishing KRAS-signaling effector genes after global expression screening has often required use of innovative experimental approaches. For example, subtractive suppression hybridization (SSH) was combined with specific signaling inhibitors of known MAPK and PI3K effector pathways to identify >200 differentially expressed genes associated with KRAS-induced malignant transformation [89]. A more recent study involved creation of a transformable, mouse cell line with an inducible, oncogenic Kras^G12D^ mutation that was used for exploring downstream signaling genes in pancreatic cancer [90]. These researchers found that KRAS activation induces an EGFR signaling loop to drive proliferation through c-MYC to play an important role for pancreatic carcinogenesis. In our study, the 2.3-fold and 3.0-fold increases in MYC and EGFR, respectively, suggest a contributory but minor role of these pathways in CAsE-PE cells.

We also performed connectivity analysis and results suggest a sizeable number of annotated relationships occur among upregulated CAsE-PE transcripts. Among the twenty-five genes with the highest number of functional interconnections, we found upregulated CAsE-PE genes that strongly support cell proliferation (in addition to KRAS) such as growth factors (EGR1, HBEGF IFG1, IGF2, LIF, VEGFA, WNT3A), transcription factors (FOS, NFE2L2, NR4A1, FOXA2) and the nuclear receptors, PPARG and PPARD. A related connectivity finding of interest was that SCNN1α, β and γ isoforms of the epithelial sodium ion channel, or ENaC, were each highly upregulated at 23-fold, 52-fold and 16-fold, respectively in CAsE-PE cells. The three SCNN1 isoforms comprise a heteromeric complex that forms a nonvoltage-gated sodium channel to regulate fluid, electrolytes and cell mobility in epithelial cells [91] and along with acid-sensing ion channels (ASICs) are part of the ENaC/degenerin family of sodium channels [92]. In prostate cells, this family of sodium channels is responsible for polarized movement of fluids and proteins for acinar lumen formation [93] but these normal ENaC activities may be repurposed for cell proliferation, migration and invasion during tumor development and progression [94]. While further work will be needed to determine if sodium channels are a direct consequence of KRAS overexpression, researchers have proposed ENaC should be targets for therapeutic intervention in various cancers [94–96].

## Conclusion

CAsE-PE cells comprise a well-studied *in vitro* human epithelial malignant transformation model produced by continuous low level arsenite exposure. Expression analysis showed >3,000 upregulated and about 4,000 downregulated transcripts that support increased cell growth, motility, survival and tumorigenic pathways consistent with prostate cancer. KRAS transcript was highly expressed in CAsE-PE cells, consistent with our prior work [25, 38], and is driven in part by an increase in KRAS copy number. Genomic analysis revealed a KRAS allelic imbalance with high expression of a mutated transcript carrying oncogenic mutations at codons 12 and 59 and many silent mutations, accompanied by relatively low expression of a wt allele. KRAS4a is the predominant transcript variant in CAsE-PE cells compared to KRAS4b in parental RWPE-1 epithelia and compared to normal primary prostate epithelium. These data are consistent with KRAS driven proliferation pathways found in spontaneous tumors or cell lines. Future work will focus on how KRAS becomes amplified in the *in vitro* arsenic transformation model to provide further insight into this important environmental carcinogen.

## Material and methods

### Cells and cell culture

RWPE-1 cells were originally isolated from normal human prostate epithelial cells and immortalized with a single copy of human papillomavirus 18 (HPV 18), are diploid, and have been shown to be non-tumorigenic, showing anchorage dependence and no tumors in nude mice [34, 97]. RWPE-1 cells were originally obtained from the Webber lab [97] and were grown in K-SFM containing 50 μg/ml bovine pituitary extract, 5 ng/ml epidermal growth factor, supplemented with antibiotic–antimycotic mixture. Cells were incubated at 37°C in a humidified atmosphere containing 5% CO2 and passaged weekly. CAsE-PE cells were originally developed in our lab by Waalkes [35] and maintained by Tokar and colleagues [38] after continuous exposure of parental cells to 5 μM sodium arsenite where media was changed 3 times per week for 29–30 weeks. RWPE-1 (control) and CAsE-PE cells (arsenic transformed) were harvested from three different cultures and frozen at -80°C until DNA and RNA isolation.

### DNA and RNA isolation

Genomic DNA and RNA were isolated from frozen cell pellets using RNeasy or DNeasy spin columns (Qiagen Valencia, CA, USA). For RNA-seq, RNA libraries were created from three independent isolates of RWPE-1 and CAsE-PE cells. Starting with 1 μg total RNA, polyA-tailed mRNA was isolated by oligo(dT) and fragmented by adaptive focused acoustic energy (Covaris Inc., MA, USA). A random hexamer primed, cDNA library of nucleotide sequences (350 bp median fragment size) was created from which millions of short DNA 100bp paired end reads were generated. Sequencing was performed on an Illumina HiSeq2000 instrument (Illumina, San Diego, CA, USA).

DNA was sheared in a Covaris instrument (S220, Woburn, MA) from which a DNA library was created by priming with random hexamers and nucleotide sequences (400 bp median fragment size) were created from 100bp DNA reads in paired-end orientation. Sequencing was performed in two lanes per cell line on an Illumina HiSeq2000 instrument (San Diego, CA) for which each lane produced 240 million raw reads.

### Bioinformatic analysis: Alignment of paired end reads

RNA samples were sequenced by the standard Illumina protocol to create raw sequence files (.fastq files) which underwent quality control analysis using FastQC (http://www.bioinformatics.babraham.ac.uk/projects/fastqc/). Quality Control (QC) plots are provided in the supplementary information (S1 Fig). We aligned the quality checked reads to the hg19 of human genome using TopHat version 2.0.11 (parameters: max gap length: 5, max mismatches: 5, max edit distance: 5, read realign edit dist: 0, mate inner distance: 250)[50].

Aligned reads were converted to UCSC genome browser tracks and uploaded to the browser to allow for visual inspection of normalized signal at any genomic location. The UCSC browser tracks contain RPKM (Reads Per Kilobase per Million reads) normalized read counts. Deep sequencing fastq data files for expression analysis and alignment are stored in the Sequence Read Archive (SRA) under Study Accession Nos. SUB4913016 and PRJNA514436.

### Analysis of differential gene expression

Differentially expressed genes (DEGs) were identified using Cuffdiff version 2.2.0 [98, 99]. Differential expression analysis was done on the human RefSeq transcriptome.

### Pathway analysis

The set of differentially expressed genes from RNA-Seq was generated (2-fold change, q<0.05) and used as input for Ingenuity Pathway Analysis (IPA) software (licensed use of Ingenuity Systems, www.ingenuity.com). Core Analysis was performed (Fig 1) to determine top canonical and disease pathways populated by differential expression. The significance value associated with overrepresented pathways measures the likelihood of an association between an experimental gene set and a reference gene set for a specific process or pathway. The p-value is calculated with the right-tailed Fisher’s Exact Test. Ingenuity (IPA) uses public databases (e.g. HumanCyc) and performs in-house curation to formulate and update signaling pathways and gene transcript and product interactions.

In our study, we focused on using differentially upregulated transcripts to build IPA ‘Grow’ pathways and IPA Connectivity analysis since there was more enrichment of annotated interactions for cell-proliferation and transformation processes compared to using down-regulated genes. The IPA Build pathway function used the ‘Grow’ feature (Fig 2) to show annotated KRAS relationships with other upregulated RefSeq DEGs (filtered to top 500 DEGs to focus on the most critical relationships). The Connect feature displayed Ingenuity-curated relationships among the top upregulated 1,000 DEGs (gene limit of connectivity algorithm), shown in Fig 3 and results were displayed in a circle graph to reduce visual complexity of gene-to-gene relationships. The number of connections to each DEG were counted as either a regulatory ability (upstream arrows ‘From’ each DEG) or as a regulated gene with a downstream connection (arrows ‘To’ regulated DEG). The higher the number of connections, the more overall regulatory importance was inferred about connections for a specific differentially expressed gene. The number of regulatory connections for each DEG (either ‘To’ or ‘From’ connections to other genes) were rank ordered for the top 25 ‘To’ (Downstream) or ‘From’ (Upstream) connections and appear in large font in Fig 3. Bolded genes in black font in the Total Scoring tab in S4 Table were those common genes that were shared in the Top 25 ‘To’ (Downstream) or ‘From’ (Upstream) connections.

### KRAS sequencing and CNV

A series of exon primers were designed from the DNA sequences of KRAS (NM033360.3) that were anchored in exons or surrounding intron-exon boundaries (S6 Table). Each exon was PCR amplified from gDNA (Phusion high fidelity DNA polymerase, NEB Inc, Ipswich ME), gel-purified and Sanger sequenced. Some experiments also involved amplification across multiple exons (exons 1–2, 2–3, 1-4a, 2-4a, and 3-4a) and the resulting amplicons were also gel purified prior to sequencing. Exon 1 was specifically examined for DNA sequence variation (e.g. codons 12 and 13) using primers at the intron-exon junctions, using wt and mutant primers (S6 Table), using sequence data derived from DNA-seq of CAsE-PE samples. CAsE-PE samples had an amplicon of the expected size whereas RWPE had nonspecific amplification (S5 Fig). The amplicons of the target size from the CAsE-PE samples were gel extracted and purified for Sanger Sequencing.

Quantitative differences in KRAS from genomic DNA were determined between the two cell types using exon specific primers. Specific KRAS exons were PCR amplified by Phusion DNA polymerase and quantitated by qPCR (Model 7900HT, ABI Life Technologies, Foster City CA USA) using a FAM labeled TaqMan probe. Three separate assays were also formulated to test if introns of the *KRAS* gene were part of a duplicated genomic region containing the KRAS gene. Amplicons for each intron were 150-200bp in length with a primer set targeting intron 3 between exons 2 and 3, and a commercial TaqMan primer set for intron 5 (Assay ID Hs05280621_cn, Life Technologies #4400291) between exons 4a and 4b (S6 Table). Possible interference of a pseudogene, KRASP1 (NC_000006.12) was accounted by designing primers and probes for amplicons in the sequence specific regions of KRAS that were distinguishable from it. All Taqman targets were tested prior to qPCR with traditional PCR and Sanger sequencing to ensure the generation of a single product, which matches exactly the KRAS gene sequence (and not the pseudogene retro-KRAS). Gel separation was performed to ensure a single amplicon from PCR reactions. DNA samples from CAsE-PE and RWPE-1 were amplified in quadruplicate using each custom Taqman assay. DNA isolated from normal prostate of a male Caucasian (Corielle Institute, Camden, NJ; Cat# NA17223) was included for copy number comparison.

TaqMan Copy Number Assays were conducted simultaneously with a TaqMan Copy Number Reference Assay in a duplex real-time polymerase chain reaction (PCR). The Copy Number Assay detects the target gene or genomic sequence of interest, and the Reference Assay detects a sequence from a known two-copy gene, human RNase P H1. Copy number is determined by relative quantitation (RQ) from the ΔΔCt method. The ΔCt difference is measured between target and reference sequences, and then compared to the ΔCT values of test samples and a calibrator sample, known to have two copies. The copy number of the target is calculated as two times the relative quantity. In a copy number quantitation reaction, purified genomic DNA is combined with the TaqMan Copy Number Assay, containing two primers and a FAM dye labeled MGB probe to detect the genomic DNA target sequence. The TaqMan Copy Number Reference Assay contains two primers and a VIC dye-labeled TAMRA probe to detect the genomic DNA reference sequence. The TaqMan Genotyping Master Mix, contains AmpliTaq Gold DNA Polymerase, UP (Ultra Pure) and dNTPs required for the PCR reactions. The reference assay was run simultaneously in each well. The relative copy number of each exon for the CAsE-PE sample was compared to the RWPE-1 DNA sample using the CopyCaller v2.0 software (Applied Biosystems).

### 454 sequencing

Total RNA from RWPE, CAsE-PE and normal human prostate epithelial cells (Sciencell, Carlsbad, CA; total RNA isolated from de-identified human Caucasian primary, early passage, epithelial cultures, Cat No. 4415) was used to synthesize cDNA with a Roche cDNA Synthesis System (Cat No. 11 117 831 001) and an oligo (dT)_15_ primer. Primers for amplification targeted wt KRAS (NM_033360) and mutant KRAS sequences as shown in S6 Table and amplicons were visualized by electrophoresis (S5 Fig). CAsE-PE RNA generated primarily one amplicon using “mutantcDNA3” primers (S6 Table) while no amplification products were observed with RWPE and normal prostate samples. Two amplicons were generated from the RWPE and prostate samples, while one amplicon was primarily observed with CAsE-PE samples (gel slices were taken and extracted above this amplicon band). The two bands were consistent with the two known KRAS splice variants, KRAS4a and KRAS4b. Indexed libraries were constructed with the Roche Rapid Library Preparation Method Manual using the 454-sequencing adapter. Libraries were pooled together for sequencing on a Roche 454 GS Flx instrument. Sequences were filtered to only visualize reads between 500 and 850 bp in length to target reads that comprised the entire amplicon of each variant. Sequences were aligned to KRAS (variant 4a, NM_033360 and variant 4b, NM_004985). 454 reads were described as ‘mutant KRAS’ if the sequence contained the base changes described in Table 2 found by Sanger sequencing and 454 reads were designated as ‘wildtype’ KRAS if mutant base changes were absent upon alignment to NCBI mRNA.

## Supporting information

S1 Fig Quality metrics for RNA-seq. (PPTX)

S2 Fig Structure of KRAS variants, KRAS4a and KRAS4b. (DOCX)

S3 Fig qPCR of CNVs for KRAS exons 4a and exon 3, intron 5 and intron 3. (PPTX)

S4 Fig KRAS cross-exon transcripts in CAsE-PE shown by PCR bands in gels. (PPTX)

S5 Fig PCR amplification of wt and mutant cDNA KRAS in RWPE-1, CAsE-PE and normal human prostate cells. (PPTX)

S1 Table RNA-seq total reads and alignment summary. (XLSX)

S2 Table RNA-seq DEGs by CuffDiff for transformed CAsE-PE cells compared to control RWPE-1 cells. (XLSX)

S3 Table 34 gene references for KRAS annotations. (XLSX)

S4 Table DEG gene interaction tables for data in Fig 3. (XLS)

S5 Table DAVID analysis. (XLSX)

S6 Table Primers for PCR, Sanger sequencing and 454 sequencing. (XLSX)

S7 Table KRASP1 and KRAS alignment. (DOCX)

S8 Table 454 sequencing read counts. (XLSX)
