# Supplementary material for: Arsenite malignantly transforms human prostate epithelial cells in vitro by gene amplification of mutated KRAS
Source: PLoS One. 2019 Apr 22;14(4):e0215504. doi: 10.1371/journal.pone.0215504 (PMC6476498; doi:10.1371/journal.pone.0215504)
Supplement: S1 Fig — (PPTX) [file pone.0215504.s001.pptx]

## Slide 1
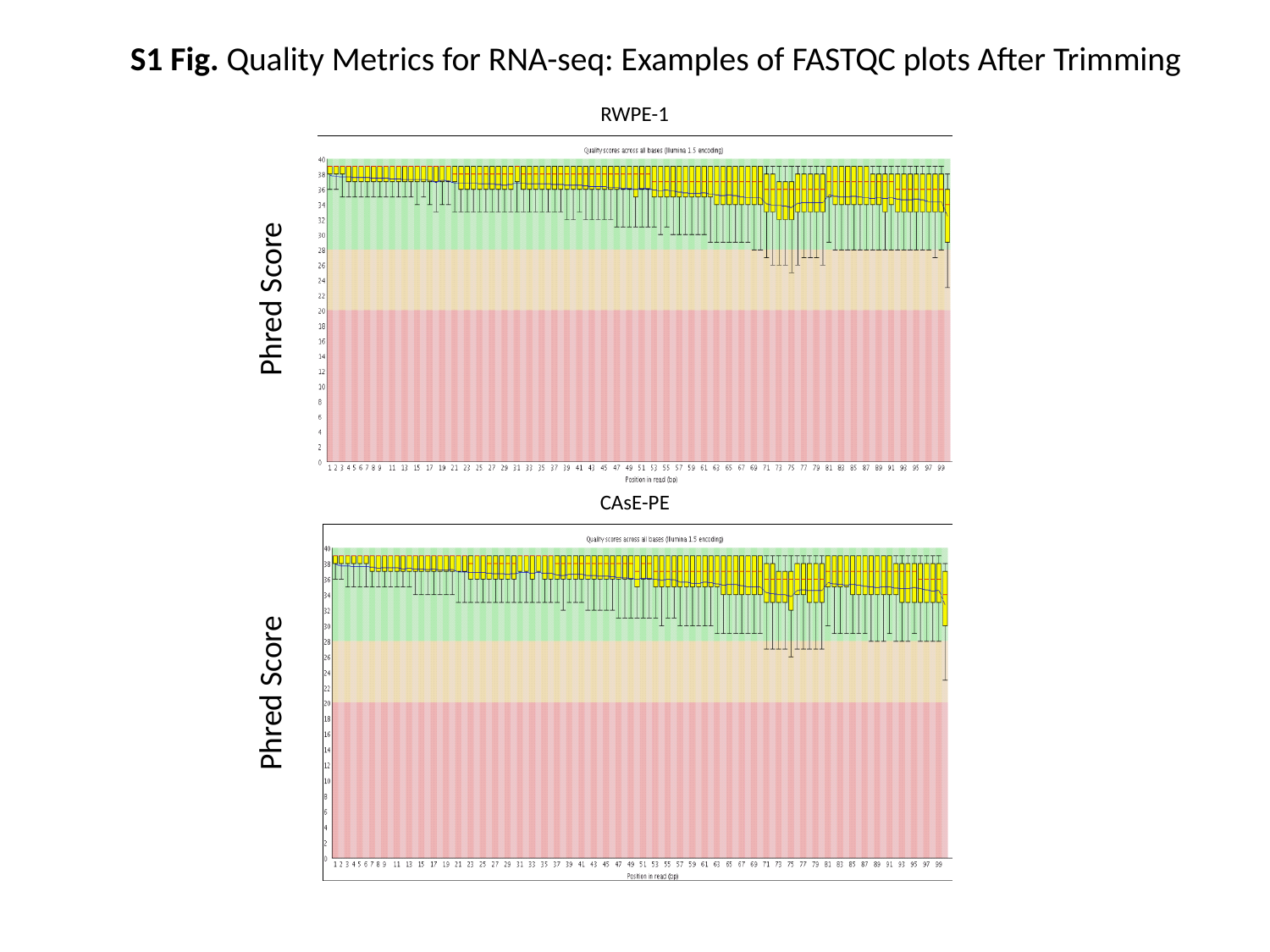

S1 Fig. Quality Metrics for RNA-seq: Examples of FASTQC plots After Trimming
RWPE-1
Phred Score
CAsE-PE
Phred Score
