# Supplementary material for: Arsenite malignantly transforms human prostate epithelial cells in vitro by gene amplification of mutated KRAS
Source: PLoS One. 2019 Apr 22;14(4):e0215504. doi: 10.1371/journal.pone.0215504 (PMC6476498; doi:10.1371/journal.pone.0215504)
Supplement: S2 Fig — (DOCX) [file pone.0215504.s002.docx]

**2S Fig.** Human Gene Structure and Sequence of KRAS Variants


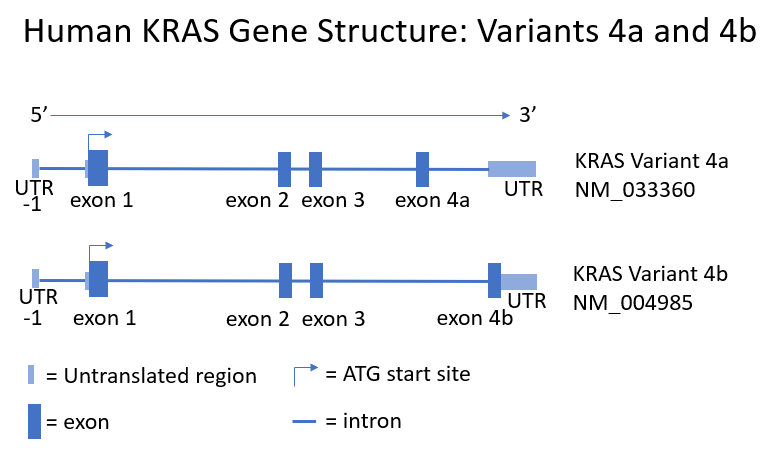


**2S Figure legend.**

The human KRAS gene structures for variants 4A and 4B are shown above. The RefSeq sequence for each sequence shown below with transitions between exons shown in light blue font. The translated products are also shown with the unique portions of the translation product shown as underlined amino acids for the 4A and 4B variants. The comparative alignment of variants 4A and 4B using the MUSCLE algorithm shows the invariant region and unique sequences for each variant.

NM_033360

>KRAS_Variant_4A_570nt

ATGACTGAATATAAACTTGTGGTAGTTGGAGCTGGTGGCGTAGGCAAGAGTGCCTTGACGATACAGCTAATTCAGAATCATTTTGTGGACGAATATGATCCAACAATAGAGGATTCCTACAGGAAGCAAGTAGTAATTGATGGAGAAACCTGTCTCTTGGATATTCTCGACACAGCAGGTCAAGAGGAGTACAGTGCAATGAGGGACCAGTACATGAGGACTGGGGAGGGCTTTCTTTGTGTATTTGCCATAAATAATACTAAATCATTTGAAGATATTCACCATTATAGAGAACAAATTAAAAGAGTTAAGGACTCTGAAGATGTACCTATGGTCCTAGTAGGAAATAAATGTGATTTGCCTTCTAGAACAGTAGACACAAAACAGGCTCAGGACTTAGCAAGAAGTTATGGAATTCCTTTTATTGAAACATCAGCAAAGACAAGACAGAGAGTGGAGGATGCTTTTTATACATTGGTGAGGGAGATCCGACAATACAGATTGAAAAAAATCAGCAAAGAAGAAAAGACTCCTGGCTGTGTGAAAATTAAAAAATGCATTATAATGTAA

>KRAS Variant 4A_189aa

MTEYKLVVVGAGGVGKSALTIQLIQNHFVDEYDPTIEDSYRKQVVIDGETCLLDILDTAGQEEYSAMRDQYMRTGEGFLCVFAINNTKSFEDIHHYREQIKRVKDSEDVPMVLVGNKCDLPSRTVDTKQAQDLARSYGIPFIETSAKTRQRVEDAFYTLVREIRQYRLKKISKEEKTPGCVKIKKCIIM

ATGACTGAAT ATAAACTTGT GGTAGTTGGA GCTGGTGGCG TAGGCAAGAG 50

TGCCTTGACG ATACAGCTAA TTCAGAATCA TTTTGTGGAC GAATATGATC 100

CAACAATAGA GGATTCCTAC AGGAAGCAAG TAGTAATTGA TGGAGAAACC 150

TGTCTCTTGG ATATTCTCGA CACAGCAGGT CAAGAGGAGT ACAGTGCAAT 200

GAGGGACCAG TACATGAGGA CTGGGGAGGG CTTTCTTTGT GTATTTGCCA 250

TAAATAATAC TAAATCATTT GAAGATATTC ACCATTATAG AGAACAAATT 300

AAAAGAGTTA AGGACTCTGA AGATGTACCT ATGGTCCTAG TAGGAAATAA 350

ATGTGATTTG CCTTCTAGAA CAGTAGACAC AAAACAGGCT CAGGACTTAG 400

CAAGAAGTTA TGGAATTCCT TTTATTGAAA CATCAGCAAA GACAAGACAG 450

AGAGTGGAGG ATGCTTTTTA TACATTGGTG AGGGAGATCC GACAATACAG 500

ATTGAAAAAA ATCAGCAAAG AAGAAAAGAC TCCTGGCTGT GTGAAAATTA 550

AAAAATGCAT TATAATGTAA

NM_004985

>KRAS_Variant_4B_567nt

ATGACTGAATATAAACTTGTGGTAGTTGGAGCTGGTGGCGTAGGCAAGAGTGCCTTGACGATACAGCTAATTCAGAATCATTTTGTGGACGAATATGATCCAACAATAGAGGATTCCTACAGGAAGCAAGTAGTAATTGATGGAGAAACCTGTCTCTTGGATATTCTCGACACAGCAGGTCAAGAGGAGTACAGTGCAATGAGGGACCAGTACATGAGGACTGGGGAGGGCTTTCTTTGTGTATTTGCCATAAATAATACTAAATCATTTGAAGATATTCACCATTATAGAGAACAAATTAAAAGAGTTAAGGACTCTGAAGATGTACCTATGGTCCTAGTAGGAAATAAATGTGATTTGCCTTCTAGAACAGTAGACACAAAACAGGCTCAGGACTTAGCAAGAAGTTATGGAATTCCTTTTATTGAAACATCAGCAAAGACAAGACAGGGTGTTGATGATGCCTTCTATACATTAGTTCGAGAAATTCGAAAACATAAAGAAAAGATGAGCAAAGATGGTAAAAAGAAGAAAAAGAAGTCAAAGACAAAGTGTGTAATTATGTAA

>KRAS Variant 4B_188aa

MTEYKLVVVGAGGVGKSALTIQLIQNHFVDEYDPTIEDSYRKQVVIDGETCLLDILDTAGQEEYSAMRDQYMRTGEGFLCVFAINNTKSFEDIHHYREQIKRVKDSEDVPMVLVGNKCDLPSRTVDTKQAQDLARSYGIPFIETSAKTRQGVDDAFYTLVREIRKHKEKMSKDGKKKKKKSKTKCVIM

ATGACTGAAT ATAAACTTGT GGTAGTTGGA GCTGGTGGCG TAGGCAAGAG 50

TGCCTTGACG ATACAGCTAA TTCAGAATCA TTTTGTGGAC GAATATGATC 100

CAACAATAGA GGATTCCTAC AGGAAGCAAG TAGTAATTGA TGGAGAAACC 150

TGTCTCTTGG ATATTCTCGA CACAGCAGGT CAAGAGGAGT ACAGTGCAAT 200

GAGGGACCAG TACATGAGGA CTGGGGAGGG CTTTCTTTGT GTATTTGCCA 250

TAAATAATAC TAAATCATTT GAAGATATTC ACCATTATAG AGAACAAATT 300

AAAAGAGTTA AGGACTCTGA AGATGTACCT ATGGTCCTAG TAGGAAATAA 350

ATGTGATTTG CCTTCTAGAA CAGTAGACAC AAAACAGGCT CAGGACTTAG 400

CAAGAAGTTA TGGAATTCCT TTTATTGAAA CATCAGCAAA GACAAGACAG 450

GGTGTTGATG ATGCCTTCTA TACATTAGTT CGAGAAATTC GAAAACATAA 500

AGAAAAGATG AGCAAAGATG GTAAAAAGAA GAAAAAGAAG TCAAAGACAA 550

AGTGTGTAAT TATGTAA

>KRAS Variant 4B_188aa

MTEYKLVVVGAGGVGKSALTIQLIQNHFVDEYDPTIEDSYRKQVVIDGETCLLDILDTAGQEEYSAMRDQYMRTGEGFLCVFAINNTKSFEDIHHYREQIKRVKDSEDVPMVLVGNKCDLPSRTVDTKQAQDLARSYGIPFIETSAKTRQGVDDAFYTLVREIRKHKEKMSKDGKKKKKKSKTKCVIM

**MUSCLE Alignment of KRAS Variant4a and Variant4b**

KRAS_Variant_4A_570nt ATGACTGAATATAAACTTGTGGTAGTTGGAGCTGGTGGCGTAGGCAAGAGTGCCTTGACG

KRAS_Variant_4B_567nt ATGACTGAATATAAACTTGTGGTAGTTGGAGCTGGTGGCGTAGGCAAGAGTGCCTTGACG

************************************************************

KRAS_Variant_4A_570nt ATACAGCTAATTCAGAATCATTTTGTGGACGAATATGATCCAACAATAGAGGATTCCTAC

KRAS_Variant_4B_567nt ATACAGCTAATTCAGAATCATTTTGTGGACGAATATGATCCAACAATAGAGGATTCCTAC

************************************************************

KRAS_Variant_4A_570nt AGGAAGCAAGTAGTAATTGATGGAGAAACCTGTCTCTTGGATATTCTCGACACAGCAGGT

KRAS_Variant_4B_567nt AGGAAGCAAGTAGTAATTGATGGAGAAACCTGTCTCTTGGATATTCTCGACACAGCAGGT

************************************************************

KRAS_Variant_4A_570nt CAAGAGGAGTACAGTGCAATGAGGGACCAGTACATGAGGACTGGGGAGGGCTTTCTTTGT

KRAS_Variant_4B_567nt CAAGAGGAGTACAGTGCAATGAGGGACCAGTACATGAGGACTGGGGAGGGCTTTCTTTGT

************************************************************

KRAS_Variant_4A_570nt GTATTTGCCATAAATAATACTAAATCATTTGAAGATATTCACCATTATAGAGAACAAATT

KRAS_Variant_4B_567nt GTATTTGCCATAAATAATACTAAATCATTTGAAGATATTCACCATTATAGAGAACAAATT

************************************************************

KRAS_Variant_4A_570nt AAAAGAGTTAAGGACTCTGAAGATGTACCTATGGTCCTAGTAGGAAATAAATGTGATTTG

KRAS_Variant_4B_567nt AAAAGAGTTAAGGACTCTGAAGATGTACCTATGGTCCTAGTAGGAAATAAATGTGATTTG

************************************************************

KRAS_Variant_4A_570nt CCTTCTAGAACAGTAGACACAAAACAGGCTCAGGACTTAGCAAGAAGTTATGGAATTCCT

KRAS_Variant_4B_567nt CCTTCTAGAACAGTAGACACAAAACAGGCTCAGGACTTAGCAAGAAGTTATGGAATTCCT

************************************************************

KRAS_Variant_4A_570nt TTTATTGAAACATCAGCAAAGACAAGACAGAGAGTGGAGGATGCTTTTTATACATTGGTG

KRAS_Variant_4B_567nt TTTATTGAAACATCAGCAAAGACAAGACAGGGTGTTGATGATGCCTTCTATACATTAGTT

****************************** * ** ** ***** ** ******** **

KRAS_Variant_4A_570nt AGGGAGATCCGACAATACAGATTGAAAAAAATCAGCAAAGAAG---AAAAGACTCCTGGC

KRAS_Variant_4B_567nt CGAGAAATTCGAAAACATAAA---GAAAAGATGAGCAAAGATGGTAAAAAGAA-------

* ** ** *** ** * * * **** ** ******** * ******

KRAS_Variant_4A_570nt TGTGTGAAAATTAA---------AAAATGCATTATAATGTAA

KRAS_Variant_4B_567nt -----GAAAAAGAAGTCAAAGACAAAGTGTGTAATTATGTAA

***** ** *** ** * ** ******
