# Supplementary material for: Arsenite malignantly transforms human prostate epithelial cells in vitro by gene amplification of mutated KRAS
Source: PLoS One. 2019 Apr 22;14(4):e0215504. doi: 10.1371/journal.pone.0215504 (PMC6476498; doi:10.1371/journal.pone.0215504)
Supplement: S3 Fig — (PPTX) [file pone.0215504.s003.pptx]

## Slide 1
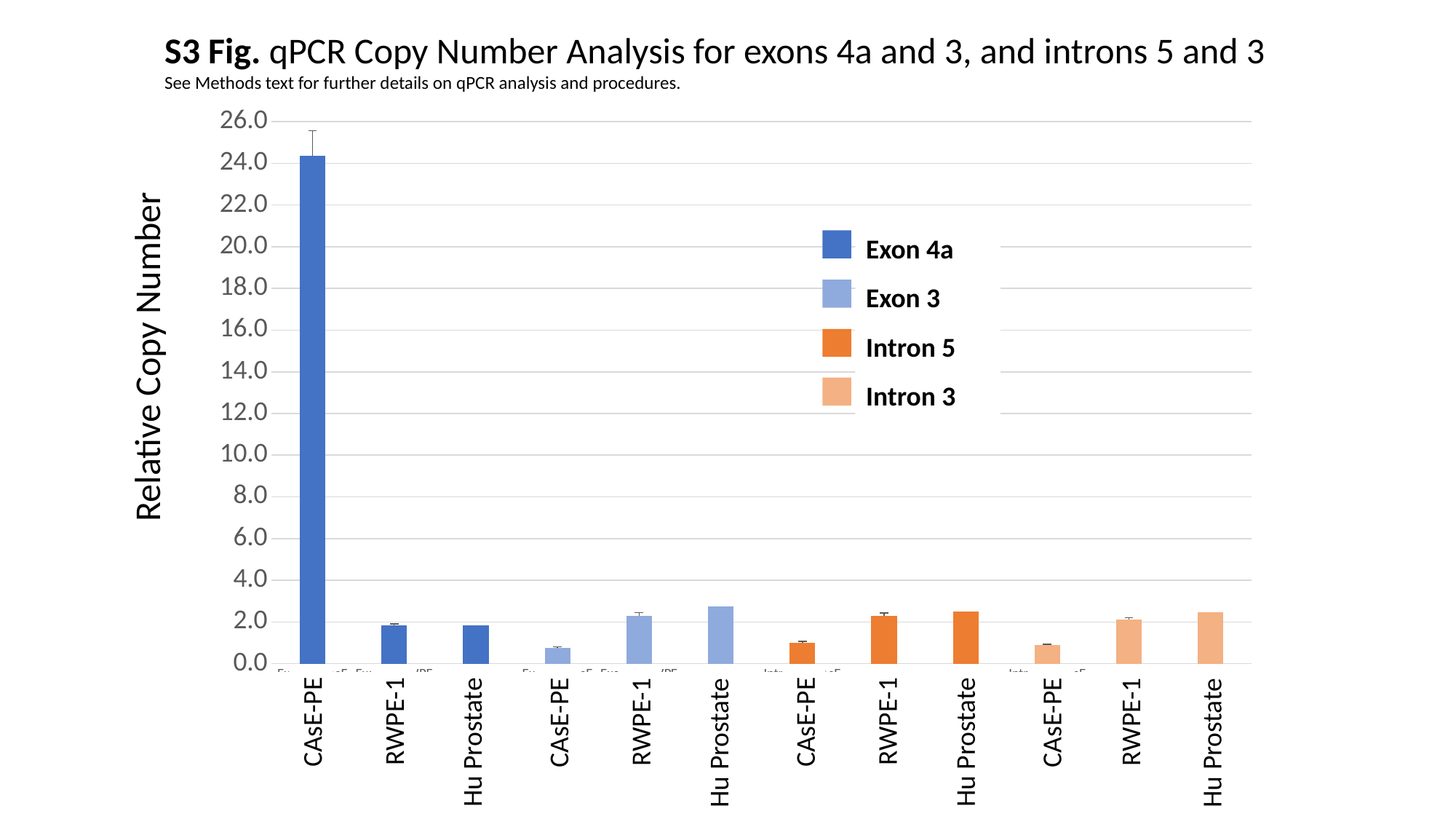

S3 Fig. qPCR Copy Number Analysis for exons 4a and 3, and introns 5 and 3
See Methods text for further details on qPCR analysis and procedures.
### Chart
| Category | |
|---|---|
| Exon 5_CAsE | 24.35 |
| Exon 5_RWPE | 1.8250000000000002 |
| Exon 5_NA17223 | 1.85 |
| Exon 4_CAsE | 0.7499999999999999 |
| Exon 4_RWPE | 2.275 |
| Exon 4_NA17223 | 2.75 |
| Intron 5_CAsE | 1.0 |
| Intron 5_RWPE | 2.3000000000000003 |
| Intron 5_NA17223 | 2.5 |
| Intron 3_CAsE | 0.9 |
| Intron 3_RWPE | 2.125 |
| Intron 3_NA17223 | 2.45 |Exon 4a
Exon 3
Intron 5
Intron 3
Relative Copy Number
RWPE-1
RWPE-1
RWPE-1
RWPE-1
CAsE-PE
CAsE-PE
CAsE-PE
CAsE-PE
Hu Prostate
Hu Prostate
Hu Prostate
Hu Prostate
