# Supplementary material for: Arsenite malignantly transforms human prostate epithelial cells in vitro by gene amplification of mutated KRAS
Source: PLoS One. 2019 Apr 22;14(4):e0215504. doi: 10.1371/journal.pone.0215504 (PMC6476498; doi:10.1371/journal.pone.0215504)
Supplement: S4 Fig — (PPTX) [file pone.0215504.s004.pptx]

## Slide 1
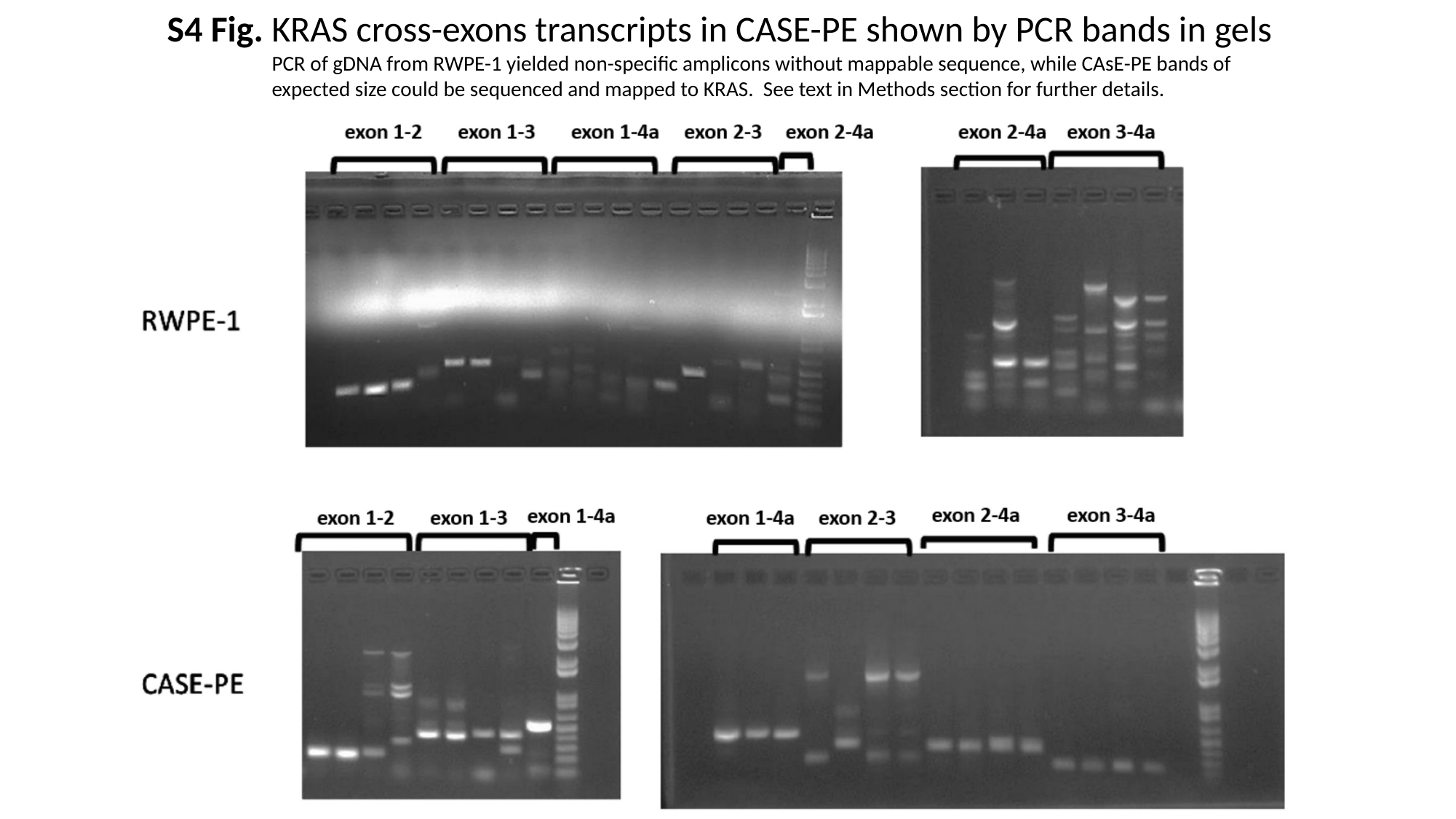

S4 Fig. KRAS cross-exons transcripts in CASE-PE shown by PCR bands in gels
PCR of gDNA from RWPE-1 yielded non-specific amplicons without mappable sequence, while CAsE-PE bands of expected size could be sequenced and mapped to KRAS. See text in Methods section for further details.
