# Supplementary material for: Arsenite malignantly transforms human prostate epithelial cells in vitro by gene amplification of mutated KRAS
Source: PLoS One. 2019 Apr 22;14(4):e0215504. doi: 10.1371/journal.pone.0215504 (PMC6476498; doi:10.1371/journal.pone.0215504)
Supplement: S5 Fig — (PPTX) [file pone.0215504.s005.pptx]

## Slide 1
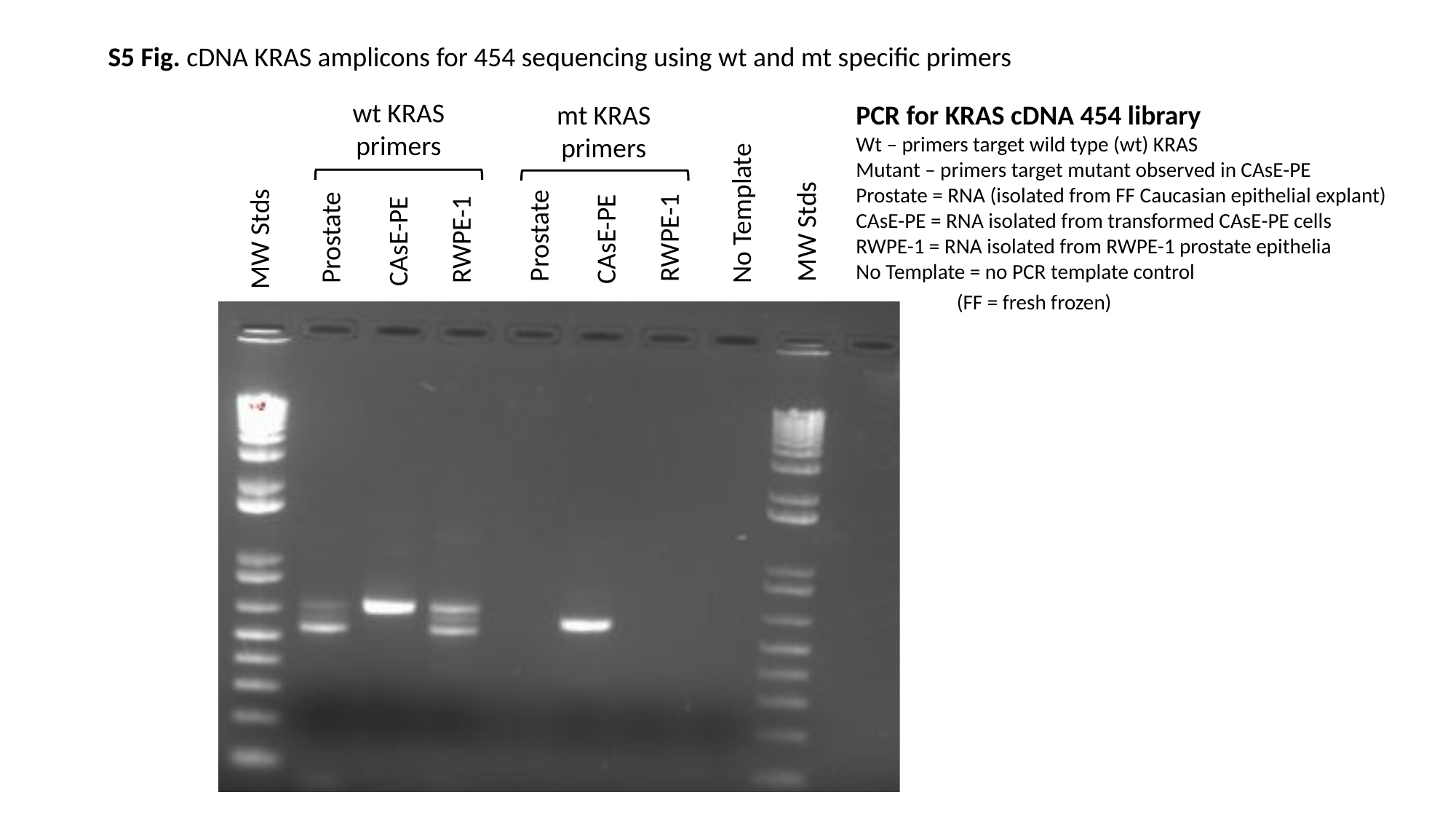

S5 Fig. cDNA KRAS amplicons for 454 sequencing using wt and mt specific primers
wt KRAS primers
mt KRAS primers
PCR for KRAS cDNA 454 library
Wt – primers target wild type (wt) KRAS
Mutant – primers target mutant observed in CAsE-PE
Prostate = RNA (isolated from FF Caucasian epithelial explant)
CAsE-PE = RNA isolated from transformed CAsE-PE cells
RWPE-1 = RNA isolated from RWPE-1 prostate epithelia
No Template = no PCR template control
No Template
MW Stds
Prostate
Prostate
MW Stds
RWPE-1
RWPE-1
CAsE-PE
CAsE-PE
(FF = fresh frozen)
