# Supplementary material for: Arsenite malignantly transforms human prostate epithelial cells in vitro by gene amplification of mutated KRAS
Source: PLoS One. 2019 Apr 22;14(4):e0215504. doi: 10.1371/journal.pone.0215504 (PMC6476498; doi:10.1371/journal.pone.0215504)
Supplement: S7 Table — (DOCX) [file pone.0215504.s012.docx]

**MUSCLE ALIGNMENT OF KRAS4A, KRAS4B, MUTANT KRAS_25Mutations (SNPs)& KRASP1**

CLUSTAL multiple sequence alignment by MUSCLE (3.8)

KRASP1_HG975407 CTGCATCATAAATTCCTAAGGAATGGAATGAGCTTTGACTGCCTTACCTTGAGAATAAGC

KRAS_VariantB_NM004985 ------------------------------------------------------------

KRAS_Mt_25SNPs ------------------------------------------------------------

KRAS_VariantA_NM033360 ------------------------------------------------------------

KRASP1_HG975407 ATATTCACGTAAAGTATATAAGACAAATACTGGTTTAGATGAAGCTCGGCCAGTACTCCC

KRAS_VariantB_NM004985 ------------------------------------------------------------

KRAS_Mt_25SNPs ------------------------------------------------------------

KRAS_VariantA_NM033360 ------------------------------------------------------------

KRASP1_HG975407 GGCCTCCGCCATTTCGGACTGGGAGCTCCGTGGCGCAGGCACTGAAGGCGGCGGCAGGGC

KRAS_VariantB_NM004985 ------------------------------------------------------------

KRAS_Mt_25SNPs ------------------------------------------------------------

KRAS_VariantA_NM033360 ------------------------------------------------------------

KRASP1_HG975407 CAGAGGCTCAGCGGCTCCCAGACCTGCTGAAAATGACTGAATATAAACTTGGCGGTAGTT

KRAS_VariantB_NM004985 --------------------------------ATGACTGAATATAAACTT-GTGGTAGTT

KRAS_Mt_25SNPs --------------------------------ATGACTGAATATAAACTT-GTGGTAGTT

KRAS_VariantA_NM033360 --------------------------------ATGACTGAATATAAACTT-GTGGTAGTT

****************** * *******

KRASP1_HG975407 GGAGCTGGTAGCGTAAGCAAAAGTGTCTTGACGATACAGCTAATTCAGAATCATTTTGTG

KRAS_VariantB_NM004985 GGAGCTGGTGGCGTAGGCAAGAGTGCCTTGACGATACAGCTAATTCAGAATCATTTTGTG

KRAS_Mt_25SNPs GGAGCT**A**GTGGCGTAGGCAAGAGTGCCTTGACGATACAGCTAATTCAGAATCATTTTGTG

KRAS_VariantA_NM033360 GGAGCTGGTGGCGTAGGCAAGAGTGCCTTGACGATACAGCTAATTCAGAATCATTTTGTG

********* ***** **** **** **********************************

KRASP1_HG975407 GACCAATATGATCCAACAATAGAGAATTCCTACAGGAAGCAAGTAGTAATTGATGGAGAA

KRAS_VariantB_NM004985 GACGAATATGATCCAACAATAGAGGATTCCTACAGGAAGCAAGTAGTAATTGATGGAGAA

KRAS_Mt_25SNPs GATGAATATGATCCTACGATAGAGGACTCCTACAGGAAACAAGTAGTAATTGATGGAGAA

KRAS_VariantA_NM033360 GACGAATATGATCCAACAATAGAGGATTCCTACAGGAAGCAAGTAGTAATTGATGGAGAA

** ********** ** ****** * *********** *********************

KRASP1_HG975407 ACCTGTCTCTTGGATATTCTTGACACAACAGGTCAAGAAGAGTACAATGCAATGA-GGAC

KRAS_VariantB_NM004985 ACCTGTCTCTTGGATATTCTCGACACAGCAGGTCAAGAGGAGTACAGTGCAATGAGGGAC

KRAS_Mt_25SNPs ACCTGTCTCTTGGATATTCTCGACACAACAGGTCAAGAGGAGTACAGTGCAATGAGGGAC

KRAS_VariantA_NM033360 ACCTGTCTCTTGGATATTCTCGACACAGCAGGTCAAGAGGAGTACAGTGCAATGAGGGAC

******************** ****** ********** ******* ******** ****

KRASP1_HG975407 CAGTACATGAGGACTGGGGGGGGGGGGGGCTTTCTTTGTGTATTTGCCATAAATAATATT

KRAS_VariantB_NM004985 CAGTACATGAGGACT------GGGGAGGGCTTTCTTTGTGTATTTGCCATAAATAATACT

KRAS_Mt_25SNPs CAGTACATGAGAACT------GGGGAGGGCTTTCTTTGTGTATTTGCCATAAATAATACT

KRAS_VariantA_NM033360 CAGTACATGAGGACT------GGGGAGGGCTTTCTTTGTGTATTTGCCATAAATAATACT

*********** *** **** ******************************** *

KRASP1_HG975407 AAATCATTTGAAGATATGCACAATTATAGAAAACAAATTAAAAGATTTAAGGACTCTGAA

KRAS_VariantB_NM004985 AAATCATTTGAAGATATTCACCATTATAGAGAACAAATTAAAAGAGTTAAGGACTCTGAA

KRAS_Mt_25SNPs AAATCATTTGAAGATATTCACCATTATAGAGAACAAATTAAAAGAGTAAAGGACTCTGAA

KRAS_VariantA_NM033360 AAATCATTTGAAGATATTCACCATTATAGAGAACAAATTAAAAGAGTTAAGGACTCTGAA

***************** *** ******** ************** * ************

KRASP1_HG975407 GATGTGCCTATGGTCCTAGTAGGAAATAAATGTGATTTGCCTTCTAGAACAGTAGACACA

KRAS_VariantB_NM004985 GATGTACCTATGGTCCTAGTAGGAAATAAATGTGATTTGCCTTCTAGAACAGTAGACACA

KRAS_Mt_25SNPs GATGTGCCTATGGTCCTAGTAGGGAATAAGTGTGACTTGCCTTCTAGAACAGTAGACACG

KRAS_VariantA_NM033360 GATGTACCTATGGTCCTAGTAGGAAATAAATGTGATTTGCCTTCTAGAACAGTAGACACA

***** ***************** ***** ***** ***********************

KRASP1_HG975407 AA--AGGCTCAGGACTTAGCAAGGAGTTAGGGAATTCCCTTTATTGAAAC--CAGCA---

KRAS_VariantB_NM004985 AAACAGGCTCAGGACTTAGCAAGAAGTTATGGAATTCCTTTTATTGAAACATCAGCAAAG

KRAS_Mt_25SNPs AAACAGGCTCAAGAGTTAGCAAGGAGTTATGGGATTCCATTCATTGAGACCTCAGCGAAG

KRAS_VariantA_NM033360 AAACAGGCTCAGGACTTAGCAAGAAGTTATGGAATTCCTTTTATTGAAACATCAGCAAAG

** ******* ** ******** ***** ** ***** ** ***** ** ****

KRASP1_HG975407 --AAGACAGGGTGTTAATGATGCCTTCTATACATTAGTTTGAGAAAT-------------

KRAS_VariantB_NM004985 ACAAGACAGGGTGTTGATGATGCCTTCTATACATTAGTTCGAGAAATTCGAAAACATAAA

KRAS_Mt_25SNPs ACAAGACAGAGAGTGGAGGATGCTTTTTATACATTGGTGAGAGAGATCCGACAGTACAGA

KRAS_VariantA_NM033360 ACAAGACAGAGAGTGGAGGATGCTTTTTATACATTGGTGAGGGAGATCCGACAATACAGA

******* * ** * ***** ** ******** ** * ** **

KRASP1_HG975407 ----AAAAGATGAGCAAAGATGGTAAGA---------AGAAGAAAAGTCA----ATGACA

KRAS_VariantB_NM004985 ---GAAAAGATGAGCAAAGATGGTAAAA---------AGAAGAAAAAGAAGTCAAAGACA

KRAS_Mt_25SNPs TTGAAAAAAATCAGCAAAGAAAAAAAGACTCCTGGCTGTGTGAAAATTAA---------A

KRAS_VariantA_NM033360 TTGAAAAAAATCAGCAAAGAAGAAAAGACTCCTGGCTGTGTGAAAATTAA---------A

**** ** ******** ** * ***** * *

KRASP1_HG975407 AAGTGTGTAATTATGTAAATACAATTAGTACTTTTTTCTTAAGGCATAGCTTAAGTAAAA

KRAS_VariantB_NM004985 AAGTGTGTAATTATGTAA------------------------------------------

KRAS_Mt_25SNPs AAATGCATTATAATGTAA------------------------------------------

KRAS_VariantA_NM033360 AAATGCATTATAATGTAA------------------------------------------

** ** * ** ******

KRASP1_HG975407 CTGGTAATTTTTATACATTACACTAAACTATTAGCATTGTTTTAGCATTACCTAATTTTT

KRAS_VariantB_NM004985 ------------------------------------------------------------

KRAS_Mt_25SNPs ------------------------------------------------------------

KRAS_VariantA_NM033360 ------------------------------------------------------------

KRASP1_HG975407 TTCCT

KRAS_VariantB_NM004985 -----

KRAS_Mt_25SNPs -----

KRAS_VariantA_NM033360 -----
